# The Soluble Guanylate Cyclase Activator BAY 58-2667 Protects against Morbidity and Mortality in Endotoxic Shock by Recoupling Organ Systems

**DOI:** 10.1371/journal.pone.0072155

**Published:** 2013-08-28

**Authors:** Benjamin Vandendriessche, Elke Rogge, Vera Goossens, Peter Vandenabeele, Johannes-Peter Stasch, Peter Brouckaert, Anje Cauwels

**Affiliations:** 1 Department for Molecular Biomedical Research, VIB, Ghent, Belgium; 2 Department of Biomedical Molecular Biology, Ghent University, Ghent, Belgium; 3 Institute of Cardiovascular Research, Bayer HealthCare, Wuppertal, Germany; Maastricht University, The Netherlands

## Abstract

Sepsis and septic shock are associated with high mortality rates and the majority of sepsis patients die due to complications of multiple organ failure (MOF). The cyclic GMP (cGMP) producing enzyme soluble guanylate cyclase (sGC) is crucially involved in the regulation of (micro)vascular homeostasis, cardiac function and, consequently, organ function. However, it can become inactivated when exposed to reactive oxygen species (ROS). The resulting heme-free sGC can be reactivated by the heme- and nitric oxide (NO)-independent sGC activator BAY 58-2667 (Cinaciguat). We report that late (+8 h) post-treatment with BAY 58-2667 in a mouse model can protect against lethal endotoxic shock. Protection was associated with reduced hypothermia, circulating IL-6 levels, cardiomyocyte apoptosis, and mortality. In contrast to BAY 58-2667, the sGC stimulator BAY 41-2272 and the phosphodiesterase 5 inhibitor Sildenafil did not have any beneficial effect on survival, emphasizing the importance of the selectivity of BAY 58-2667 for diseased vessels and tissues. Hemodynamic parameters (blood pressure and heart rate) were decreased, and linear and nonlinear indices of blood pressure variability, reflective for (un)coupling of the communication between the autonomic nervous system and the heart, were improved after late protective treatment with BAY 58-2667. In conclusion, our results demonstrate the pivotal role of the NO/sGC axis in endotoxic shock. Stabilization of sGC function with BAY 58-2667 can prevent mortality when given in the correct treatment window, which probably depends on the dynamics of the heme-free sGC pool, in turn influenced by oxidative stress. We speculate that, considering the central role of sGC signaling in many pathways required for maintenance of (micro)circulatory homeostasis, BAY 58-2667 supports organ function by recoupling inter-organ communication pathways.

## Introduction

Sepsis and septic shock are associated with mortality rates of up to 50–70% [1]. Further still, their incidence is increasing and septic shock recently became the number one cause of death in intensive care units worldwide [2], despite numerous anti-inflammatory (or “anti-mediator”) therapeutic strategies that were tested during the last few decades [3], [4]. The only mediator-targeted drug that was ever FDA approved specifically for the treatment of severe sepsis (Drotrecogin alfa), was recently withdrawn from the market [5]. Whatever the precipitating factor, septic shock is characterized by cardiovascular collapse, refractory hypotension, tissue ischemia, and cytopathic hypoxia, which can progress to multiple organ failure (MOF) and death. Because all -of the many- roads lead to MOF, regardless of the inflammatory trigger, shifting the search for therapeutics further downstream and later to the common pathway of organ failure might prove to be more beneficial.

Furthermore, *Godin and Buchman* put forward the idea that the ability of organs to adapt to changing environmental conditions is dependent on the interconnections between them [6], [7]. They postulated that the information and variability within those connections provide more information on the overall state of the system than the absolute values of individual variables at discrete time points. Thus, the systemic inflammatory response syndrome (SIRS) not only has a direct impact on cells and tissues but also on the inter-organ communication network, consisting of neural, humoral and cytokine components. Uncoupling of that network can single out organs from feedback control loops and facilitate MOF, i.e. uncoupling is a consequence of SIRS and the cause of MOF. In order to quantify the dynamic state of a complex organism, a reliable read-out is needed, such as the variability contained within a heart rate (HR) or blood pressure (BP) signal. HR and BP variability (HRV and BPV, respectively) are defined as the variation of the period between consecutive beat or diastolic intervals, respectively, which are representative for the extrinsic central nervous regulation of the intrinsic pacemaker activity of the heart and can as such be used to monitor the sympathovagal balance of the autonomic nervous system (ANS). It has been well established that indices of HRV and BPV are altered in critically ill patients [8], thus assessment of these parameters could be useful as markers for disease progression, timing, dosing and follow-up of treatment, as well as patient stratification tools.

**Figure 1:**
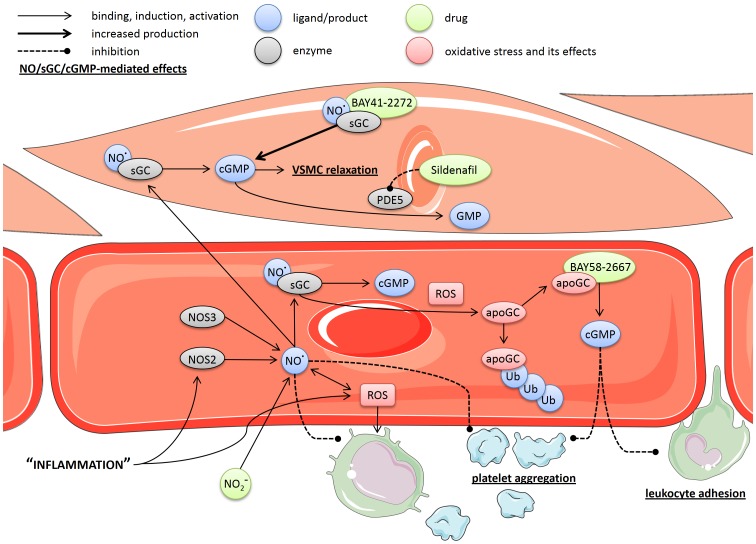
Schematic overview of drug interactions with the NO/sGC/cGMP pathway. NO produced by NOS activates sGC, resulting in the production of cGMP, and subsequent relaxation of VSMCs and inhibition of platelet aggregation and leukocyte adhesion, among others. In turn, cGMP is broken down to GMP by PDE5, which can be inhibited by Sildenafil. ROS can interact at various levels with this pathway, including facilitating the oxidation of the iron in the heme-prosthetic group of sGC. The resulting heme-free sGC (apoGC) is no longer functional and a target for rapid ubiquitin-mediated proteolytic degradation. However, BAY 58-2667 can bind heme-free sGC and reactivate cGMP production independent of NO. BAY 41-2272, on the other hand, can stimulate the activity of functional (reduced-heme) sGC synergistically with NO. Nitrite, a hypoxia selective NO donor, can also be used to stimulate this pathway selectively. No cell specific effects for the compounds or enzymes are assumed, they are shown as such for simplicity only. Arrows indicate binding, interaction or induction; bold arrows indicates increased production; dashed lines indicate an inhibitory effect; bold/underlined text indicates NO/cGMP-mediated effects; ‘apoGC’ = heme-free sGC. Figure was produced using Servier Medical Art.

Soluble guanylate cyclase (sGC) is a heterodimeric enzyme, primarily found in the α_1_β_1_ and, to a lesser extent, the α_2_β_1_ isoform. Upon binding of NO to the heme prosthetic group of the enzyme, cyclic GMP (cGMP) production is increased about 200-fold. cGMP, in turn, interacts with cGMP-activated protein kinases, cyclic nucleotide-gated channels and phosphodiesterases (PDEs), and is involved in the regulation of various physiological functions, including vasodilation, platelet aggregation, and neurotransmission [9], [10]. Previously, we showed that the hypoxia selective NO donor nitrite (NO_2_^−^) can protect against toxicity in shock via sGC-dependent signaling, which may include hypoxic vasodilation necessary to maintain microcirculation and organ function, and cardioprotection [11]. Furthermore, a study in sGCα_1_ knockout mice showed that cGMP generated by sGCα_1_β_1_ protects against cardiac dysfunction and mortality in murine inflammatory shock models [12]. In light of these findings, we decided to test the heme- and hence NO-independent sGC activator BAY 58-2667 (Cinaciguat) and the heme-dependent sGC stimulator BAY 41-2272 in a murine model of inflammatory shock (Fig. 1). ROS interact at various levels with the NO/sGC/cGMP pathway: at the level of sGC, they can oxidize the ferrous iron (Fe^2+^) in the heme prosthetic group, which can lead to release of the heme, thereby rendering the enzyme inactive and prone to ubiquitin-mediated proteolytic degradation [13]. BAY 58-2667 competes for the heme-binding motif of heme-free sGC (also known as apo-sGC), and as such protects the enzyme from proteolytic breakdown and reactivates cGMP production in the absence of NO [14]. Consequently, BAY 58-2667 is assumed to be specific for tissues that are suffering from hypoxia, cytopathic hypoxia and/or oxidative stress, and is thought to be most effective when a considerable pool of heme-free sGC is present, which can occur both in the case of chronic low-level inflammation or acute (systemic) inflammation [15], [16]. BAY 41-2272, on the other hand, will act synergistically with NO to stimulate the activity of functional (reduced-heme) sGC [17]. Documented effects of treatment with BAY 58-2667 and BAY 41-2272 in various disease models include: reducing preload and afterload, increasing cardiac output, inducing pulmonary vasodilation, reducing cardiac hypertrophy, inhibiting platelet aggregation, increasing renal blood flow and glomerular filtration rate, and lowering systemic BP [18], [19]. In addition, Sildenafil (Viagra™), a phosphodiesterase-5 (PDE5) inhibitor that inhibits breakdown of cGMP to GMP, was used as a control drug [20].

In this study, we examined the effect of post-treatment with the sGC activator BAY 58-2667 and sGC stimulator BAY 41-2272 in a model of endotoxic shock, and found that late treatment with BAY 58-2667 can protect mice from a lethal shock-inducing challenge, in contrast to BAY 41-2272. Analysis of indices of BPV indicated that improved morbidity and survival was associated with a systemic recoupling effect.

## Results and Discussion

### BAY 58-2667 protects against LPS-induced morbidity and mortality, in contrast to BAY 41-2272 and Sildenafil

**Figure 2:**
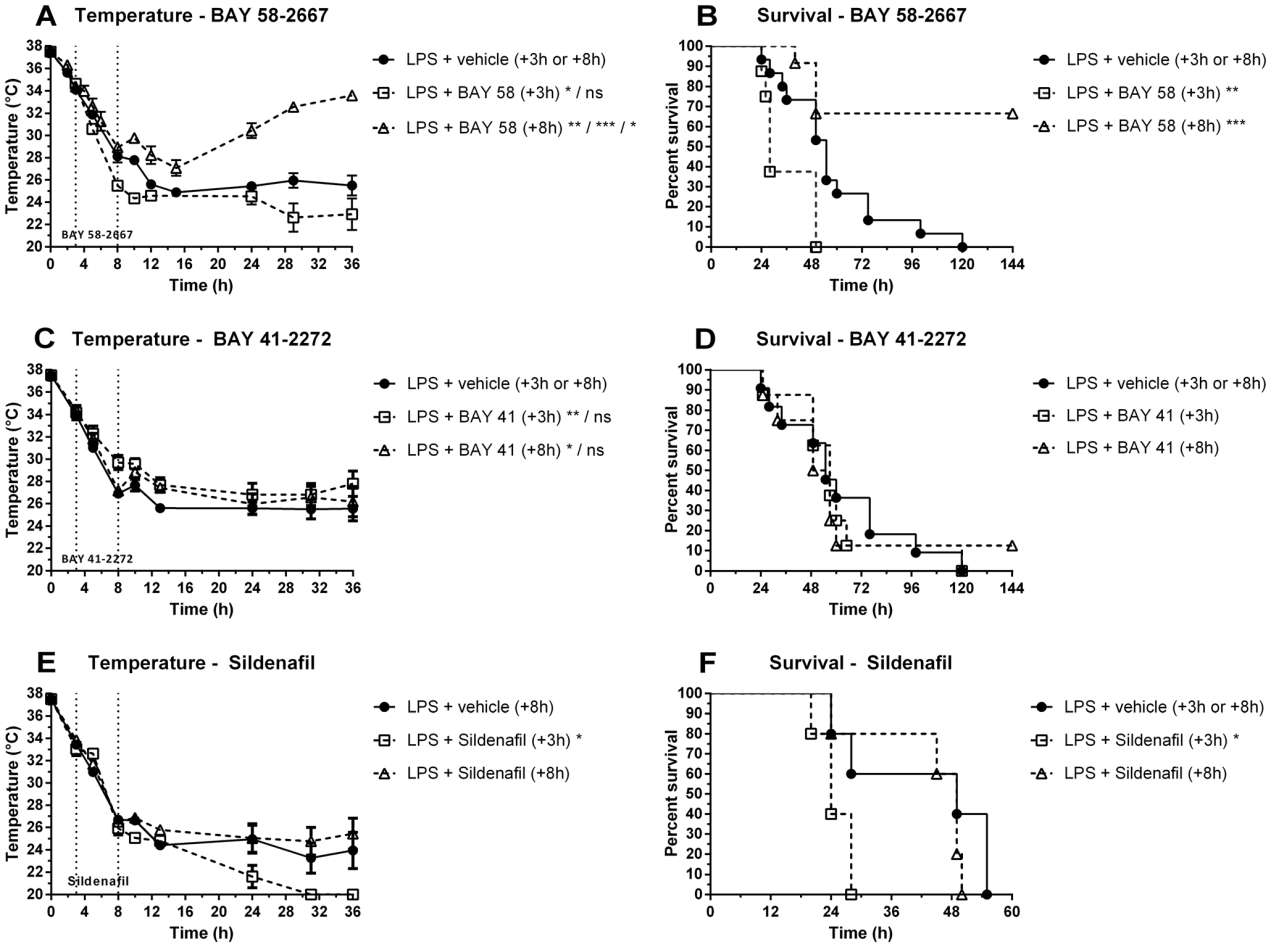
Effect of post-treatment with BAY 58-2667, BAY 41-2272, and Sildenafil on body temperature (left) and mortality (right). (A–B) WT mice were injected i.v. with 9.5–11 mg/kg LPS (*E. coli*) or 17.5 mg/kg LPS (*S. abortus* equi) at t = 0 and treated (+3 h or +8 h) i.v. with 100 µg/kg BAY 58-2667 or vehicle control (+3 h or +8 h). The combined results of three independently performed experiments are shown (n_vehicle_ = 15, n_+3 h_ = 8, n_+8 h_ = 12). (C–D) WT mice were injected i.v. with 17.5 mg/kg LPS (*S. abortus equi*) at t = 0 and treated i.v. with 100 µg/kg BAY 41-2272 (+3 h or +8 h) or vehicle control (+8 h). The combined results of two independently performed experiments are shown (n_vehicle_ = 11, n_+3 h_ = 8, n_+8 h_ = 8). (E–F) WT mice were injected i.v. with 17.5 mg/kg LPS (*S. abortus equi*) at t = 0 and treated i.v. with 1 mg/kg Sildenafil (+3 h or +8 h) or vehicle control (+8 h) (n = 5). Data are means ± SEM; temperature curves were compared to appropriate vehicle controls via repeated-measure ANOVA (see Table S1 for individual F-statistics, p- and n-values). For panel A & C, significance was calculated in relation to appropriate controls for single experiments. Survival curves were compared to controls via log-rank test for merged experiments. ****, p≤0.0001; ***, p≤0.001; **, p≤0.01; *, p≤0.05; and ns = nonsignificant.

**Figure 3:**
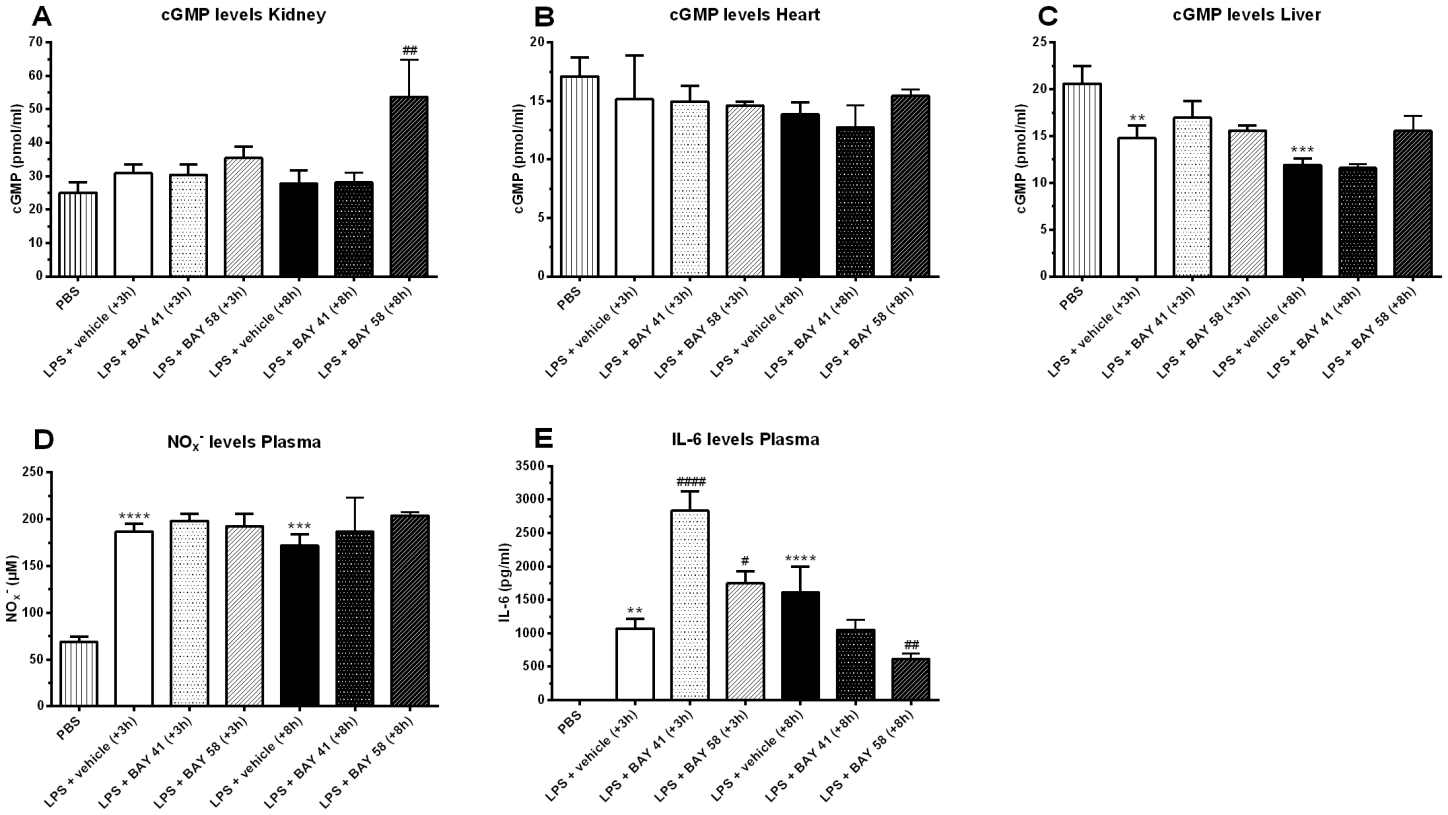
cGMP levels in kidney, heart and liver, and NO_x_^−^ and IL-6 levels in plasma. (A–C) cGMP levels in whole kidney (A), heart (B) and liver (C) homogenates, respectively, 2 h post-treatment (n = 3). (D) Circulating NO_x_^−^ levels in plasma, and (E) IL-6 levels in plasma, 2 h post-treatment (n = 3). Data are means ± SEM and comparisons were made between baseline (PBS) and LPS-challenged vehicle control animals (*), as well as LPS-challenged vehicle control and treatment groups (#) via one-way ANOVA with Fisher's LSD test. ***, p≤0.001; **, p≤0.01 and *, p≤0.05.

Late (+8 h) post-treatment with BAY 58-2667 protected against progressive hypothermia and mortality (combined survival rate of 66% versus 0% for controls) induced by a lethal dose of LPS. In contrast, early post-treatment (+3 h) exacerbated LPS-induced hypothermia and mortality (Fig. 2A–B). Post-treatment (+3 h or +8 h) with the sGC stimulator BAY 41-2272 had no or only a very small temporary positive effect on body temperature. No effect was found on survival (Fig. 2, C–D). Cyclic GMP levels in kidney were increased 2 h after late (+8 h) treatment with BAY 58-2667 (Fig. 3, A); cGMP levels in heart were not affected by treatment (Fig. 3, B). In liver, cGMP levels were downregulated due to challenge with LPS (Fig. 3, C); early or late treatment with BAY 41-2272 did not have any effect (Fig. 3, A–C). Plasma NO_x_^−^ levels, reflective for increased NO production by NOS2, were increased for all LPS-challenged animals, indicating that direct stimulation of sGC by NO was similar in all groups, independent of treatment (Fig. 3, D). Plasma IL-6 levels, a known prognostic marker for sepsis, were increased in the early (+3 h) treatment groups compared to appropriate vehicle controls, and decreased in the late (+8 h) BAY 58-2667 treatment group compared to vehicle controls, correlating with outcome (Fig. 3, E). Late (+8 h) treatment with the PDE5 inhibitor Sildenafil, used as an additional drug control, had no effect on body temperature and survival, whereas early (+3 h) treatment sensitized mice to the effects of LPS (Fig. 2, E–F), similar to early (+3 h) treatment with BAY 58-2667.

BAY 58-2667 selectively binds to heme-free sGC, thereby stabilizing the enzyme and reactivating cGMP production [16]. We showed previously that the hypoxia-selective vasodilator nitrite can protect mice against tumor necrosis factor (TNF) and LPS-induced morbidity and mortality in an sGC-dependent manner [11]. However, nitrite treatment was only able to prevent mortality in the LPS model when administered as a pretreatment and at very high doses, whereas the current study focused on later intervention by targeting a more downstream component of the pathway. We found a positive treatment effect when BAY 58-2667 was administered late (+8 h) after induction of endotoxic shock, indicating that the distribution of heme-free sGC in that window was optimal for intervention with the drug, also confirmed by the highly elevated cGMP levels in the kidney. BAY 58-2667-mediated protection is most likely a consequence of its specificity for vessels and tissues that were exposed to oxidative stress, as we did not observe similar positive effects on body temperature or survival when using the heme-dependent sGC stimulator BAY 41-2272 or the PDE5 inhibitor Sildenafil. However, early treatment with BAY 58-2667 exacerbated mortality, indicating that +3 h after LPS challenge the pool of heme-free sGC had already increased substantially, which could also explain the lack of early BAY 41-2272 effect. In addition, the sensitizing effect of BAY 58-2667 implies that reactivation of the heme-free sGC pool can have unfavorable effects when administered in the wrong time window. In a similar fashion, early (+3 h) Sildenafil treatment exacerbated mortality, indicating that bioactive cGMP was still present at that point in time, and that increasing cGMP activity by preventing its breakdown, can also sensitize mice to the effects of an LPS challenge. As this warranted further investigation, we focused on the cardiovascular system because of the pivotal role of sGC/cGMP signaling in its regulation.

### Effect of BAY 58-2667 and BAY 41-2272 treatment on cardiomyocyte apoptosis

**Figure 4:**
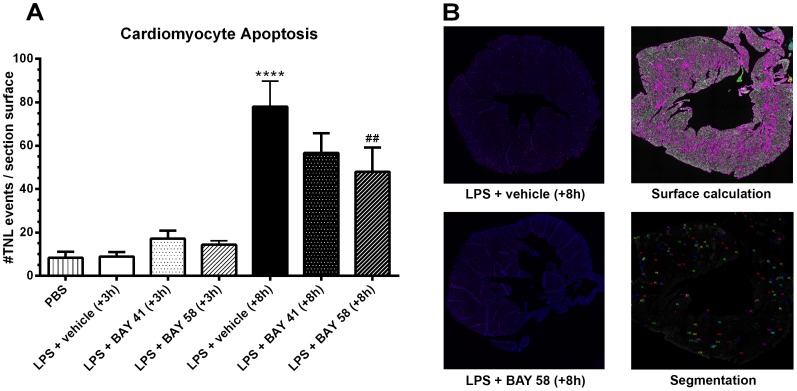
Cardiomyocyte apoptosis. (A) The number of apoptotic cells per heart section were counted and normalized over the total surface area of the tissue section, 2 h post-treatment (n = 3). (B) Representative example of whole heart section for late (+8 h) vehicle control (top-left), and late (+8 h) BAY 58-2667 treatment (bottom-left). Representative example of data processing in BD Attovision software: calculation of total surface area (top-right) and detection of TUNEL events (bottom-right). Data are means ± SEM and comparisons were made between baseline (PBS) and LPS-challenged vehicle control animals (*), and LPS-challenged vehicle control and treatment groups (#) via one-way ANOVA with Fisher's LSD test. ***, p≤0.001; **, p≤0.01 and *, p≤0.05.

Apoptotic cardiomyocytes were determined by counting the number of TUNEL positive nuclei over the entire surface area of a cross-section of the heart (Fig. 4, A): 2 h after late (+8 h) vehicle treatment, the number of apoptotic cells was increased compared to saline (PBS) and early treatment controls. Late (+8 h) treatment with BAY 58-2667 significantly reduced the amount of apoptotic cells in the heart. However, although not significant, the number of apoptotic cells was also reduced after late BAY 41-2272 treatment, hinting towards the presence of a mixed pool of oxidized- and reduced-heme sGC in the heart, 8 h post-LPS challenge. Cardiac dysfunction is one of the most important determinants of sepsis-induced mortality, both in humans and in animal models [21], [22]. Our results are corroborated by a previous study that showed that sGCα_1_-deficient mice are sensitized to inflammation-induced cardiac dysfunction, emphasizing the cardioprotective effect of sGCα_1_-derived cGMP [12]. Although cardiomyocyte apoptosis was reduced thanks to late (+8 h) BAY 58-2667 treatment, this was not reflected in increased cGMP levels in the heart (Fig. 3, B). However, samples were taken 2 h post-treatment, when cGMP levels were still highly increased in the kidney, while this increase could have been more transient in the heart. In addition, very low levels of sGC activation have been shown to elicit full level responses in smooth muscle cells [23], [24], i.e. low level stimulation of sGC and resulting small or even undetectable changes in cGMP levels can have profound cardiovascular effects. Furthermore, reduced cardiomyocyte apoptosis may not only be caused by a direct sGC-mediated effect on the heart, but could also be secondary to a more distal effect on the circulatory system.

The involvement of apoptosis in LPS-induced mortality has been corroborated by other studies [25], [26], albeit not specific to the heart. Hence, it seems likely that reduced cardiomyocyte apoptosis contributed to improved survival, and that any potentially detrimental effect of late sGC reactivation on survival -as observed for early sGC reactivation- could have been masked by this protective effect on cardiomyocyte apoptosis, among others.

### Effect of BAY 58-2667 and BAY 41-2272 treatment on systemic blood pressure and heart rate

**Figure 5:**
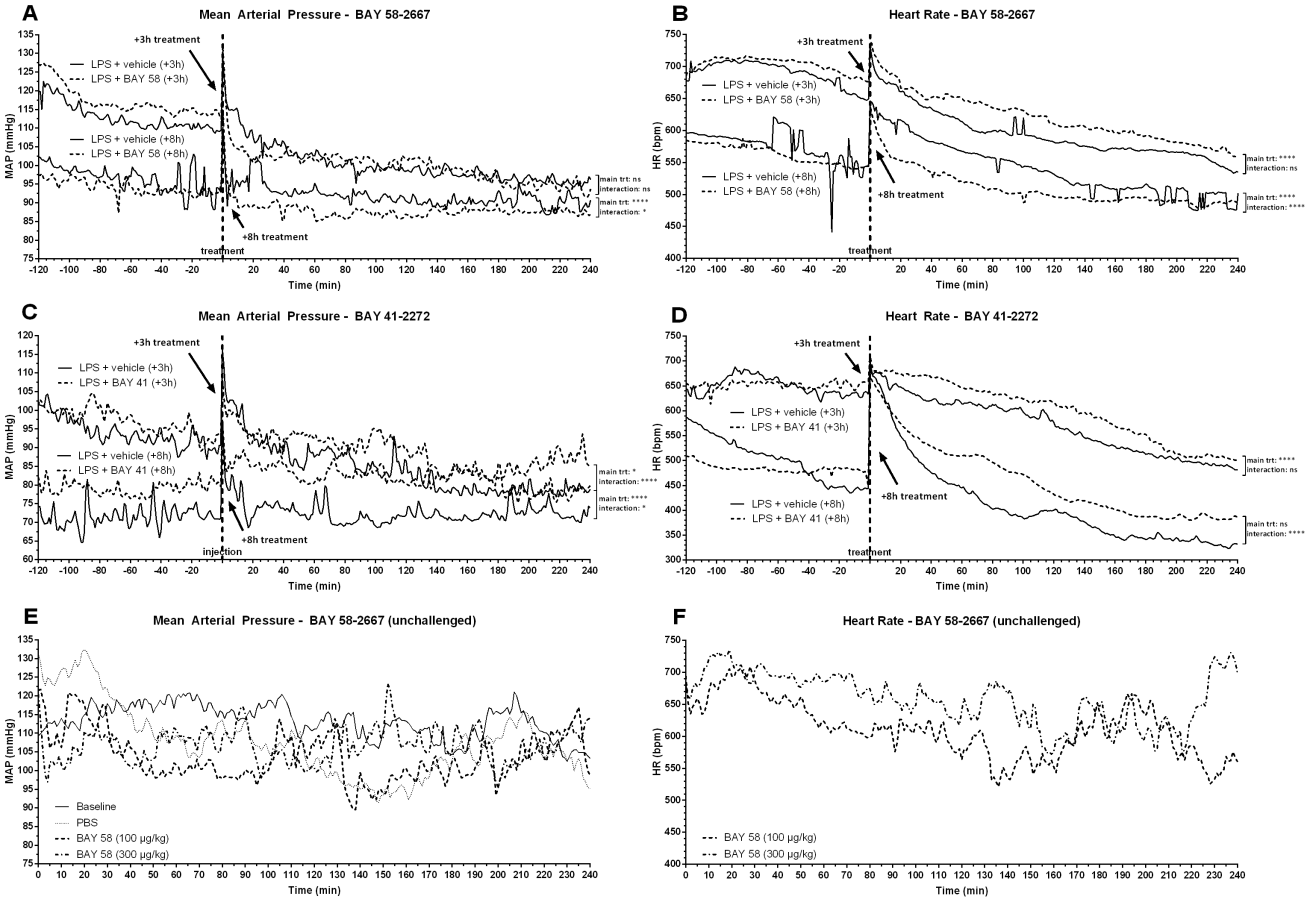
Effect of BAY 58-2667 and BAY 41-2272 treatment on hemodynamic parameters. Mean arterial pressure (MAP) and heart rate (HR) were recorded via implanted telemetry devices. (A–B) Mice were injected i.v. with 9.5–11 mg/kg LPS (*E. coli*), and treated i.v. with 100 µg/kg BAY 58-2667 (+3 h or +8 h) or vehicle control (+3 h or +8 h); 2 h pre- until 4 h post-treatment of data is shown (n = 4). (C–D) Mice were injected i.v. with 9.5–11 mg/kg LPS (*E. coli*), and treated i.v. with 100 µg/kg BAY 41-2272 (+3 h or +8 h, n = 2) or vehicle control (+3 h or +8 h, n = 1); 2 h pre- until 4 h post-treatment of data is shown. (E–F) Unchallenged mice were injected with saline (PBS), 100 µg/kg or 300 µg/kg BAY 58-2667; 4 h of data post-injection is shown (n = 4). Data are means and were compared to vehicle controls by fitting a linear mixed model (see Table S2 for fixed term statistics). ****, p≤0.0001; ***, p≤0.001; *, p≤0.05; ns = nonsignificant; trt = treatment effect and time×trt = time-treatment interaction.

Figure 5 shows hemodynamic parameters from mice that were treated with vehicle, BAY 41-2272 or BAY 58-2667, +3 h or +8 h after challenge with LPS (Fig. 5, A–D), as well as from healthy mice (Fig. 5, E–F). For LPS-challenged mice, a time window of 2 h pre- until 4 h post-treatment was analyzed. Injection of saline in healthy mice typically prompted a transient (±30 min) stress-induced increase in mean arterial pressure (MAP), while injection of BAY 58-2667 (100 µg/kg or 300 µg/kg) caused a dose-dependent drop in MAP that lasted for 15–25 min (Fig. 5, E). Shortly after the drop in MAP, a prolonged compensatory increase in HR was observed (Fig. 5, F). A phase I dose escalating study in healthy human volunteers showed that BAY 58-2667 caused a rapid decrease in systemic vascular resistance (SVR), followed by a reduction of MAP that, in turn, was followed by a compensatory increase in HR and stroke volume (SV) [27]. Thus, activation of the small pool of heme-free sGC present at baseline can have systemic effects, albeit limited. Our data in non-challenged (healthy) mice confirmed this.

For LPS-challenged animals, a brief (±40 min) lowering effect on MAP was observed immediately after early (+3 h) treatment with BAY 58-2667 (Fig. 5, A), while HR was increased for a prolonged period (Fig. 5, B). During this compensatory increased HR phase, MAP stabilized for about 90 min, indicating that early after challenge with LPS the increased HR is able to compensate for the reduction in SVR and MAP induced by BAY 58-2667. Early (+3 h) treatment with BAY 41-2272 did not have an acute effect on MAP (Fig. 5, C), while HR was increased compared to vehicle controls for about 2.5 h, similar to BAY 58-2667 (Fig. 5, D). Thus, the transient drop in MAP specific to early (+3 h) BAY 58-2667 treatment, could be responsible for the increased mortality observed in this treatment group, whether or not combined with a possible lack of effect on cardiomyocyte apoptosis. In contrast, for late BAY 58-2667 (+8 h) treatment an effect on MAP was found that lasted for approximately 2.5 h (Fig. 5, A), while HR was decreased in the same time window (Fig. 5, B). For late (+8 h) BAY 41-2272 treatment, MAP and HR appeared to be different from vehicle controls (Fig. 5, C). However, the difference in MAP was already present before treatment and is thus not likely caused by a drug or vehicle effect. Also the delayed onset of the increase in HR post-treatment, is more likely to be related to variation in the response to LPS than a direct drug effect.

The fact that the effect of late (+8 h) BAY 58-2667 treatment on MAP lasted longer compared to early (+3 h) treatment, indicates that the pool of heme-free sGC was increasing during the progression of endotoxic shock, and that reactivation of this pool had a larger effect on SVR. However, the decrease in MAP following late (+8 h) treatment was not associated with an elevated HR; on the contrary, HR was lower compared to controls. Exactly how the combination of reduced MAP and HR are linked to improved survival is unclear, but it implies that the baroreceptor reflex may be malfunctioning, a documented effect of LPS toxicity [28], thereby failing to translate the drop in MAP into a compensatory tachycardia, which appeared to be still functional +3 h after LPS challenge. Alternatively, BAY 58-2667 could have had a direct effect on HR, masking the effect of the reflex arc. Nevertheless, successful treatment was associated with increased peripheral body temperature (Fig. 2, A), indicative for improved perfusion.

### BAY 58-2667 recouples communication between the autonomic nervous system and the heart

As a readout of systemic organ function, we analyzed BPV. The numerous feedback loops inherent to any kind of biological system are essential to allow a dynamic system to respond to changing environmental conditions, and prevent excessive “mode-locking” [29]. A direct consequence is that, rather than the individual values of different variables within the system, the interconnections between those variables are more representative for the health status of the organism. Disturbing such a system, for instance by a massive inflammatory insult, will cause uncoupling of the organs resulting in organ failure due to lack of inter-organ communication and inability to respond to environmental input [6], [7]. The variability inherent in the oscillatory signals that form the basis of these interconnections can be measured in certain biological signals, of which HR and BP are most straightforward to obtain. If BAY 58-2667 indeed had a systemic effect on inter-organ communication, this should be reflected in the variability imposed by the sympathetic and parasympathetic branches of the ANS on the BP signal.

**Figure 6:**
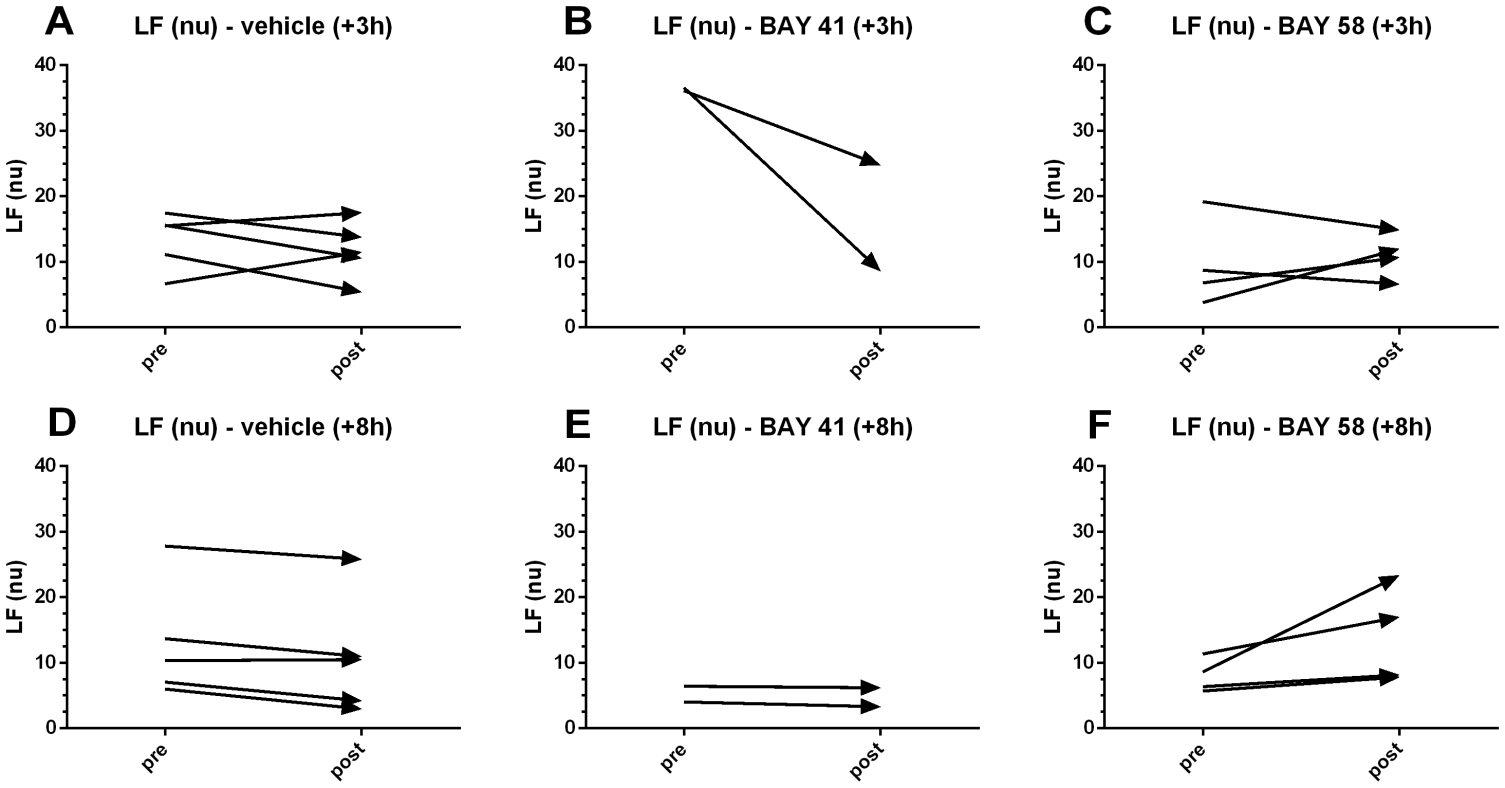
Effect of BAY 58-2667 and BAY 41-2272 treatment on LF (nu). Normalized low frequency (LF (nu)) values for 40 min pretreatment were compared to 40 min post-treatment values and plotted as changing trends over time for vehicle +3 h (A), BAY 41-2272 +3 h (B), BAY 58-2667 +3 h (C), vehicle +8 h (D), BAY 41-2272 +8 h (E), and BAY 58-2667 +8 h (F).

Two indices of variability were analyzed: the low frequency band, obtained via fast Fourier transformation (FFT) of the original time series; and the scaling factors, calculated via detrended fluctuation analysis (DFA). The normalized LF (nu) band was quantified for BP data in the pre- and post-treatment interval (40 min each). For early (+3 h) BAY 58-2667 treatment and corresponding vehicle control groups, the change in LF (nu) was randomly distributed across different animals (Fig. 6, A & C), whereas the trends for early BAY 41-2272 were negative (Fig. 6, B). In contrast, an increase in the normalized LF band was observed for late (+8 h) BAY 58-2667 treatment for all animals (Fig. 6, F), while decreasing trends were observed for the corresponding vehicle (+8 h) control and BAY 41-2272 treated animals (Fig. 6, D–E). Increased activity in the normalized LF (nu) band indicates mainly an increase in sympathetic tone. Thus, successful treatment with BAY 58-2667 was reflected in a temporary increase in sympathetic signaling from the ANS to the heart, not observed for BAY 41-2272 treatment. These results seem counterintuitive, as an increase in sympathetic signaling is usually associated with an increase in HR, while we observed a prolonged decrease in HR for late (+8 h) BAY 58-2667 treatment (Fig. 5, B). This suggests again that late after LPS challenge, the baroreceptor reflex is indeed no longer functional, resulting in bradycardia as a net secondary effect of late (+8 h) BAY 58-2667 treatment, despite the presence of increased power in the LF band.

**Figure 7:**
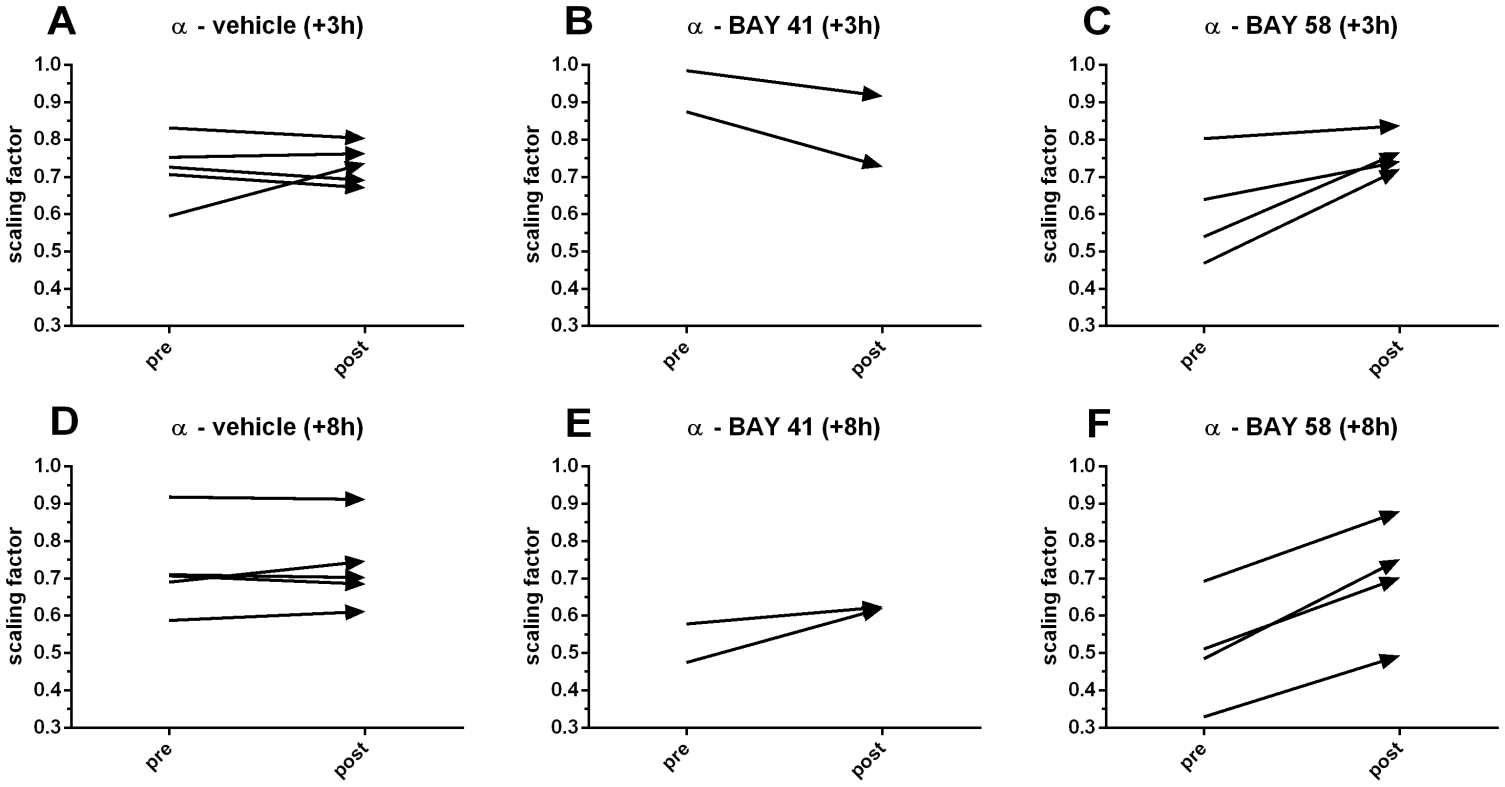
Effect of BAY 58-2667 and BAY 41-2272 treatment on scaling factors. Scaling factors (α) for 40 min pretreatment were compared to 40 min post-treatment values and plotted as changing trends over time for vehicle +3 h (A), BAY 41-2272 +3 h (B), BAY 58-2667 +3 h (C), vehicle +8 h (D), BAY 41-2272 +8 h (E), and BAY 58-2667 +8 h (F).

Scaling factors quantify the fractal properties of a time series and were increased after treatment with BAY 58-2667 in both the +3 h (Fig. 7, C) and +8 h (Fig. 7, F) treatment group to values closer to 1.0, the physiological optimum, while trends were randomly distributed for both vehicle control groups (Fig. 7, A & D). Trends were negative for early (+3 h) and slightly positive for late (+8 h) treatment with BAY 41-2272 (Fig. 7, B & E), although the latter was not reflected in the LF (nu) trends. Thus, the BAY 58-2667-mediated effect was most pronounced for late treatment, but also present in the early treatment group, indicating that despite the lack of positive effect on survival, early treatment with BAY 58-2667 also had some effect on neurological regulation of the heart. However, scaling factors are not limited to activity in a specific spectral band, as is the case for LF (nu), suggesting that the observed increase in the power of the LF band may be a more important determinant for survival.

## Conclusion

Summarized, our data show that protective post-treatment with the sGC activator BAY 58-2667 is associated with reduced hypothermia and mortality, reduced cardiomyocyte apoptosis, reduced plasma IL-6 levels, and a reduction in HR. Late treatment with BAY 41-2272 also had some minor (nonsignificant) effects on cardiomyocyte apoptosis and IL-6 levels, despite a complete lack of any beneficial effect on body temperature or survival. Because sGC is a major regulator of (micro)circulatory flow [30], stabilization of its function may improve perfusion and decrease hypoxia and cytopathic hypoxia, subsequently supporting organ function and survival. Since the pool of heme-free sGC is increasing during the progression of endotoxic shock, the beneficial effects of BAY 58-2667 compared to the lack of effect of BAY 41-2272 on survival could be attributed to its selectivity for tissues and vessels that were exposed to high levels of oxidative stress. To substantiate this hypothesis, we determined indices of BPV that are reflective for heart-ANS coupling and found that early and late BAY 58-2667 treatment both influence communication between the ANS and the heart, but protective (+8 h) treatment is specifically associated with increased activity in the LF (nu) band, indicative for increased sympathetic signaling.

In conclusion, our results demonstrate the pivotal role of the NO/sGC/cGMP axis in endotoxic shock. Stabilization of sGC function with BAY 58-2667 can prevent mortality induced by LPS when given in the correct treatment window, which probably depends on the dynamics of the heme-free sGC pool, in turn influenced by ROS. We speculate that the effect of BAY 58-2667 on (micro)circulatory homeostasis supports cardiac and organ function by recoupling inter-organ communication pathways. Selective stimulation of this pathway could be a new treatment paradigm for sepsis and septic shock, as was already shown with the hypoxia selective NO donor nitrite [11]. Treatment strategies aimed at sGC can interfere more downstream in the common path to MOF, allowing more time between diagnosis and start of treatment. However, selectivity and spatiotemporal targeting appear to be extremely important to avoid deleterious effects, warranting further investigation in more complex models. In addition, there is a need for biomarkers that can help in identifying the optimal treatment window for intervention with sGC activators in those models. One of the most pressing issues with regard to SIRS patient management, is the lack of validated biomarkers that allow reliable and early diagnosis, one of the most important determinants of survival [31]. Indices of variability hold promise as prognostic tools as they are known to be altered in critically ill patients, and HR and BP are routinely measured in the ICU. Coupling experimental treatment approaches to a biomarker that allows follow-up of treatment response, could allow for a more efficient translation of results between various disease models, as well as to the clinic.

## Materials and Methods

### Mice

Female C57BL/6J mice were purchased from Janvier (France). All mice were housed in temperature-controlled, individually ventilated cages in an SPF facility with 14/10 h light/dark cycles, food and water *ad libitum*, and used at 10–16 weeks of age.

### Ethics Statement

All experiments were approved by the animal ethics committee of the Faculty of Sciences of Ghent University (Belgium) and performed according to its guidelines.

### Reagents and injections

All reagents were dissolved in sterile PBS and injected intravenously, unless stated otherwise. Phenol extracted *E. coli* LPS (serotype O111:B4 or O55:B5) and phenol extracted *S. abortus equi* LPS were purchased from Sigma (St. Louis, MO (USA)) and administered at 9.5–11 mg/kg (*E. coli*) or 17.5 mg/kg (*S. abortus equi*) to induce endotoxic shock. BAY 58-2667 (Cinaciguat) and BAY 41-2272 were administered as a post-treatment at 100 µg/kg, dissolved in vehicle (20% diethylene glycol monoethyl ether (DGME, Sigma), 20% Cremophor EL (Fluka, Sigma) and 60% sterile PBS). Sildenafil citrate was purchased from Tocris Bioscience (Bristol, UK) and administered as a post-treatment at 1 mg/kg in 0.8% dimethyl sulfoxide (DMSO, Sigma) in sterile PBS.

### Body temperature and hemodynamic measurements

Rectal body temperatures were recorded on an electronic thermometer (C28K, Comark Electronics; Littlehampton, UK). BP, HR, and activity were measured continuously in conscious mice via radiotelemetry (PA-C10 probes, Data Sciences International, St. Paul, MN) as previously described [32].

### Plasma NO_x_^−^ and IL-6 levels

Plasma was prepared from blood collected 2 h post-treatment via cardiac puncture after terminal anesthesia with xylazine/ketamine and immediately flash frozen in liquid nitrogen (n = 3). Plasma concentrations of NO_2_^−^ and NO_3_^−^ (collectively NO_x_^−^) were determined via the Griess method as previously described [32]. Plasma concentrations of IL-6 were determined via 7TD1 cell bioassay, as previously described [33].

### Cyclic GMP assay

Kidneys, hearts and livers were isolated 2 h post-treatment and snap frozen in liquid nitrogen (n = 3). cGMP levels in whole organ homogenates were measured via enzyme-immunoassay (Monoclonal anti-cGMP EIA kit, NewEast Biosciences, King of Prussia, PA (USA)) as per the manufacturer's instructions.

### Immunohistochemical staining of apoptotic cardiomyocytes

Hearts were isolated 2 h post-treatment and fixed in 4% paraformaldehyde (n = 3). After dehydration, paraffin embedding and sectioning, the tissue was stained with a Terminal deoxynucleotidyl transferase dUTP Nick End Labeling (TUNEL) method (*In Situ* Cell Death Detection Kit, TMR Red, Roche Diagnostics, Vilvoorde, Belgium) as per the manufacturer's instructions. Next, sections were counterstained with DAPI nuclear staining and imaged (10× magnification) on a BD Pathway 855 automated imaging system (BD Biosciences, Erembodegem, Belgium). The number of TUNEL events was counted and normalized over the entire tissue surface area with BD Attovision analysis software.

### Analysis of BP variability

Two broad categories of analysis methods exist for analysis of variability in biological time series: (1) linear methods, e.g. frequency domain analysis, and (2) nonlinear methods, e.g. detrended fluctuation analysis (DFA). For the frequency domain, fast Fourier transformation (FFT) was used to divide the power spectrum in discrete frequency bands that can be linked to activity in specific branches of the ANS. The spectral power in the low frequency (LF) band is assumed to reflect both sympathetic and parasympathetic tone, while the high frequency (HF) band reflects vagal (parasympathetic) tone [34], [35]. Nonlinear methods are expected to extract more relevant physiological information considering the inherent complexity of biological signals. DFA quantifies the fractal properties of a time series [36], [37]. A scaling factor α of 1.0 indicates the presence of long-range correlations, characteristic for proper communication between the ANS and regulation of the heart rhythm. Higher or lower values indicate breakdown of correlations, indicative for failure of communication. Appropriate BP traces were imported into ADI Labchart Pro v7.3 (ADInstruments GmbH, Spechbach, Germany). BP variability parameters were calculated using the HRV module v1.4.2. Frequency domain parameters were calculated with the following settings: FFT size 1024, Window Welch, Overlap ½, Max frequency 5 Hz, Frequency bands 0.15<LF<1.5<HF<5 Hz. Low and high frequency bands (LF and HF, respectively) were normalized over the very low frequency (VLF) component subtracted from total power according to 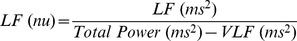. Next, diastolic interval time series were exported and spline corrected using the *hrspline* and *ardeglch* functions (courtesy of [38]) in Matlab v7.13 (The MathWorks Inc., Natick, MA (USA)). The fractal properties of the spline corrected time series were analyzed using the detrended fluctuation algorithm (DFA) [36], [37]. Next, the scaling factor α was calculated by fitting a linear trend through the DFA result on a log-log plot.

### Statistical Analysis

Statistical analysis was performed with GraphPad Prism 6.01 (GraphPad Software, La Jolla, CA (USA)) and SAS software 9.2 (SAS Institute Inc., Cary, NC (USA)). Temperature curves were compared to appropriate controls via repeated-measure ANOVA. A survival function was estimated with the Kaplan-Meier estimator to assess the marginal effect of treatment on time of death. Survival curves of treatment groups were compared to appropriate controls using the log-rank (Mantel-Cox) test. For *ex vivo* analysis, baseline levels were compared to vehicle controls, and vehicle controls to appropriate treatment groups using one-way ANOVA. Means were compared with a Fisher's LSD test. Longitudinal data analysis was performed by fitting the following linear mixed model: effect = β_1_+β_2_t+β_3_T+β_4_tT where t is time, T is treatment, and tT is the interaction term. Times of measurement were equally spaced and various ways of modeling the correlation structure (simple, unstructured, autoregressive order 1, and compound symmetry) were compared using the residual maximum likelihood (REML) method in the mixed model framework as implemented in SAS. Selection of the best model fit was based on likelihood ratio test (LRT) statistics and the Aikake Information coefficient (AIC). All appropriate diagnostics were carefully examined. Values are means ± SEM, unless stated otherwise.

## Supporting Information

Table S1 Repeated-measure ANOVA. F-statistics, p-values and n-values for Figure 2. Statistics were calculated for separate (non-merged) experiments where appropriate. ****, p≤0.0001; ***, p≤0.001; **, p≤0.01; *, p≤0.05 and ns = nonsignificant.
(DOCX)

Table S2 Linear mixed model. Fixed term F-statistics for Figure 5 A–D. Longitudinal data analysis was performed by fitting the following linear mixed model: β_1_+β_2_t+β_3_T+β_4_tT where t is time, T is treatment, and tT is the interaction term. Times of measurement were equally spaced and a ‘simple’ model for the correlation structure was used in the residual maximum likelihood (REML) framework as implemented in SAS. ****, p≤0.0001; **, p≤0.01; *, p≤0.05 and ns = nonsignificant.
(DOCX)
