# Supplementary material for: The Soluble Guanylate Cyclase Activator BAY 58-2667 Protects against Morbidity and Mortality in Endotoxic Shock by Recoupling Organ Systems
Source: PLoS One. 2013 Aug 28;8(8):e72155. doi: 10.1371/journal.pone.0072155 (PMC3756074; doi:10.1371/journal.pone.0072155)
Supplement: Table S1 — Repeated-measure ANOVA. F-statistics, p-values and n-values for Figure 2. Statistics were calculated for separate (non-merged) experiments where appropriate. ****, p≤0.0001; ***, p≤0.001; **, p≤0.01; *, p≤0.05 and ns = nonsignificant. (DOCX) [file pone.0072155.s001.docx]

**Table S1. Repeated-measure ANOVA.**

| **Fig2A** | **time** | **treatment effect** | **Pr > F** | |  | **n** |
| --- | --- | --- | --- | --- | --- | --- |
|  | +3h | F(1, 8) = 6.708 | 0.0321 | | * | 3 |
|  | +3h | F(1, 10) = 2.810 | 0.1246 | | ns | 5 |
|  | +8h | F(1, 6) = 31.42 | 0.0014 | | ** | 4 |
|  | +8h | F(1, 8) = 39.49 | 0.0002 | | *** | 3 |
|  | +8h | F(1, 10) = 6.007 | 0.0342 | | * | 5 |
| **Fig2C** | **time** | **treatment effect** | **Pr > F** |  | | **n** |
|  | +3h | F(1, 8) = 20.87 | 0.0018 | ** | | 5 |
|  | +3h | F(1, 4) = 0.12 | 0.7466 | ns | | 3 |
|  | +8h | F(1, 8) = 10.15 | 0.0129 | * | | 5 |
|  | +8h | F(1, 4) = 0.44 | 0.5421 | ns | | 3 |
| **Fig2E** | **time** | **treatment effect** | **Pr > F** |  | | **n** |
|  | +3h | F(1, 8) = 6.453 | 0.0347 | * | | 5 |
|  | +8h | F(1, 8) = 0.93 | 0.3632 | ns | | 5 |

F-statistics, p-values and n-values for Figure 2. Statistics were calculated for separate (non-merged) experiments where appropriate. ****, p ≤ 0.0001; ***, p ≤ 0.001; **, p ≤ 0.01; *, p ≤ 0.05 and ns = nonsignificant.
