# Supplementary material for: The Soluble Guanylate Cyclase Activator BAY 58-2667 Protects against Morbidity and Mortality in Endotoxic Shock by Recoupling Organ Systems
Source: PLoS One. 2013 Aug 28;8(8):e72155. doi: 10.1371/journal.pone.0072155 (PMC3756074; doi:10.1371/journal.pone.0072155)
Supplement: Table S2 — Linear mixed model. Fixed term F-statistics for Figure 5 A–D. Longitudinal data analysis was performed by fitting the following linear mixed model: β1+β2t+β3T+β4tT where t is time, T is treatment, and tT is the interaction term. Times of measurement were equally spaced and a ‘simple’ model for the correlation structure was used in the residual maximum likelihood (REML) framework as implemented in SAS. ****, p≤0.0001; **, p≤0.01; *, p≤0.05 and ns = nonsignificant. (DOCX) [file pone.0072155.s002.docx]

**Table S2. Linear mixed model.**

|  | **+3h comparison** |  |  |  |  |
| --- | --- | --- | --- | --- | --- |
| **Fig5A** | **fixed term** | **F statistic** | **den df** | **Pr > F** |  |
|  | time | 516.87 | 1837 | < 0.0001 | **** |
|  | treatment | 3.5 | 1837 | 0.0615 | ns |
|  | time x trt | 2.25 | 1837 | 0.1337 | ns |
|  |  |  |  |  |  |
|  | **+8h comparison** |  |  |  |  |
|  | **fixed term** | **F statistic** | **den df** | **Pr > F** |  |
|  | time | 44.11 | 1884 | < 0.0001 | **** |
|  | treatment | 25.05 | 1884 | < 0.0001 | **** |
|  | time x trt | 5.67 | 1844 | 0.0173 | * |
|  |  |  |  |  |  |
|  | **+3h comparison** |  |  |  |  |
| **Fig5B** | **fixed term** | **F statistic** | **den df** | **Pr > F** |  |
|  | time | 764.62 | 1836 | < 0.0001 | **** |
|  | treatment | 29.49 | 1836 | < 0.0001 | **** |
|  | time x trt | 0.28 | 1836 | 0.5965 | ns |
|  |  |  |  |  |  |
|  | **+8h comparison** |  |  |  |  |
|  | **fixed term** | **F statistic** | **den df** | **Pr > F** |  |
|  | time | 565.81 | 1881 | < 0.0001 | **** |
|  | treatment | 107.91 | 1881 | < 0.0001 | **** |
|  | time x trt | 30.76 | 1881 | < 0.0001 | **** |
|  | **+3h comparison** |  |  |  |  |
| **Fig5C** | **fixed term** | **F statistic** | **den df** | **Pr > F** |  |
|  | time | 153.17 | 293 | < 0.0001 | **** |
|  | treatment | 4.06 | 293 | 0.0449 | * |
|  | time x trt | 23.01 | 293 | < 0.0001 | **** |
|  |  |  |  |  |  |
|  | **+8h comparison** |  |  |  |  |
|  | **fixed term** | **F statistic** | **den df** | **Pr > F** |  |
|  | time | 69.14 | 293 | < 0.0001 | **** |
|  | treatment | 69.86 | 293 | < 0.0001 | **** |
|  | time x trt | 5.57 | 293 | 0.0189 | * |
|  | **+3h comparison** |  |  |  |  |
| **Fig5D** | **fixed term** | **F statistic** | **den df** | **Pr > F** |  |
|  | time | 282.60 | 293 | < 0.0001 | **** |
|  | treatment | 36.07 | 293 | < 0.0001 | **** |
|  | time x trt | 0.34 | 293 | 0.5632 | ns |
|  |  |  |  |  |  |
|  | **+8h comparison** |  |  |  |  |
|  | **fixed term** | **F statistic** | **den df** | **Pr > F** |  |
|  | time | 1420.09 | 293 | < 0.0001 | **** |
|  | treatment | 0.49 | 293 | 0.4843 | ns |
|  | time x trt | 93.87 | 293 | < 0.0001 | **** |

Fixed term F-statistics for Figure 5 A-D. Longitudinal data analysis was performed by fitting the following linear mixed model: β_1_ + β_2_t + β_3_T + β_4_tT where t is time, T is treatment, and tT is the interaction term. Times of measurement were equally spaced and a ‘simple’ model for the correlation structure was used in the residual maximum likelihood (REML) framework as implemented in SAS. ****, p ≤ 0.0001; **, p ≤ 0.01; *, p ≤ 0.05 and ns = nonsignificant.
